# Machine learning-based prediction model for 28-day mortality in acute kidney injury patients with liver cirrhosis: A MIMIC-IV database analysis

**DOI:** 10.1371/journal.pone.0328662

**Published:** 2025-09-08

**Authors:** Luyu Chai, Yuxiang Zhou, Nan Zhou, Yao Xiao, Renqi Pang

**Affiliations:** 1 Hainan Lecheng Institute of Real World Study, Qionghai, Hainan Province, China; 2 Rehabilitation Department, Zhoushan Guanghua Hospital, Zhoushan, Zhejiang Province, China; 3 Centre for Cancer Research, M. Kandiah Faculty of Medicine and Health Sciences, UTAR: Universiti Tunku Abdul Rahman, China; 4 Jiangxi College of Traditional Chinese Medicine, Fuzhou, Jiangxi, China; 5 Guangzhou Tianhe District Hospital of Chinese Medicine, Guangzhou, China; Institute of Clinical and Experimental medicine: Institut klinicke a experimentalni mediciny, CZECHIA

## Abstract

**Background:**

Acute kidney injury (AKI) in patients with liver cirrhosis represents a significant clinical challenge with high mortality rates. This study aimed to develop and validate a machine learning-based prediction model for 28-day mortality in AKI patients with liver cirrhosis using the MIMIC-IV database.

**Methods:**

This retrospective study analyzed data from 4,168 AKI patients, including 601 with concurrent liver cirrhosis, from the MIMIC-IV database. Patient selection followed strict inclusion and exclusion criteria. The study implemented comprehensive data preprocessing, including feature normalization and selection through Recursive Feature Elimination. Multiple machine learning algorithms were evaluated, with model performance assessed through ROC curves, calibration curves, and precision-recall analysis. SHAP analysis was conducted to interpret feature contributions to mortality prediction.

**Results:**

The liver cirrhosis group demonstrated distinct clinical characteristics, including significantly lower age (median 60 vs 70 years, p < 0.001) and higher disease severity scores (SOFA 11 vs 8 points) compared to non-cirrhotic patients. Survival analysis confirmed significantly lower 28-day survival probability in the cirrhosis group (Log-rank test, χ2 = 46.5, p < 0.001). The Random Forest model achieved optimal performance with an AUC of 0.85 and precision-recall area of 0.81. SHAP analysis identified pH, anion gap, and total CO2 as the most significant predictive factors, with notable interaction effects among these indicators.

**Conclusion:**

This study successfully developed a machine learning model for predicting 28-day mortality in AKI patients with liver cirrhosis. The model demonstrated superior clinical decision-making value compared to traditional scoring systems, particularly in moderate-risk threshold intervals. The findings emphasize the crucial role of acid-base balance indicators in mortality risk assessment, providing valuable insights for clinical intervention strategies.

## Introduction

Acute kidney injury (AKI) is a clinical syndrome characterized by rapid deterioration of renal function, manifesting as an increase in serum creatinine levels exceeding 26.5 μmol/L from baseline within 48 hours or a 1.5-fold increase from baseline within 7 days [1]. The global annual incidence reaches 13.2 million cases, with a mortality rate of 23.9%. In the population aged 40–75 years, the incidence rate is 13.4%, with a higher prevalence in males than females [2]. AKI, as a significant complication of liver cirrhosis, substantially increases the risk of adverse outcomes [3]. Studies indicate that AKI patients with liver cirrhosis demonstrate a 52% higher in-hospital mortality rate compared to those with AKI alone, primarily through mechanisms including portal hypertension, hepatorenal syndrome, systemic inflammatory response, and coagulation dysfunction, which collectively exacerbate renal impairment [4]. Current research data reveals that approximately 21.3% of hospitalized AKI patients present with varying degrees of liver cirrhosis, and both the incidence and mortality rates of AKI demonstrate a significant upward trend with increasing severity of liver cirrhosis [5].

The Medical Information Mart for Intensive Care (MIMIC) is a critical care research data platform established through collaboration between the Laboratory for Computational Physiology at Massachusetts Institute of Technology and Beth Israel Deaconess Medical Center [6]. The current latest version, MIMIC-IV, encompasses clinical data from over 50,000 critically ill patients between 2008−2019, containing 76,540 structured data variables covering multiple dimensions including demographic characteristics, vital signs, laboratory tests, medication orders, nursing records, imaging examinations, and clinical events [7]. The database implements rigorous de-identification procedures, utilizing SHA-256 hash encryption algorithms and relative time shifting for all clinical information to ensure patient privacy protection. The unique advantages of MIMIC database lie in its fine data granularity (minimum sampling interval of 1 minute), comprehensive information dimensions (containing complete ICU hospitalization processes), reliable data quality (validated through multiple verifications), and complete open access (following PhysioNet data use agreement). In the field of kidney disease research, this database provides an ideal foundation platform for developing machine learning prediction models due to its standardized data structure and comprehensive clinical information.

This research develops machine learning prediction models based on the MIMIC database to quantitatively assess 28-day mortality risk in AKI patients with liver cirrhosis. The study incorporates AKI patients diagnosed in MIMIC-IV version 2.2 database, establishing prediction models for both groups with and without liver cirrhosis through analysis of multidimensional data including clinical laboratory indicators, organ function scores, and therapeutic interventions. The research aims to provide medical teams with individualized risk assessment tools to optimize treatment strategy selection and prognosis evaluation for these high-risk patients. The establishment of this prediction model addresses the current gap in standardized prognostic assessment tools for AKI patients with liver cirrhosis, providing more precise quantitative evidence for clinical decision-making.

## Method

### Data selection

Study subjects were selected from the MIMIC-IV database through strict inclusion and exclusion criteria. Inclusion criteria comprised: [1] adult patients with first hospitalization (age ≥ 18 years); [2] AKI patients diagnosed according to ICD-9 and ICD-10 disease codes; [3] liver cirrhosis patients diagnosed according to ICD-9 and ICD-10 disease codes. Exclusion criteria included: [1] hospitalization duration less than 24 hours; [2] intensive care unit stay less than 24 hours; [3] incomplete clinical data (missing items >20%); [4] patients with pre-existing end-stage renal disease or receiving renal replacement therapy; [5] patients with sepsis complication. All timestamps in the MIMIC database are provided as blinded relative time. No personally identifying patient information is included. The data utilized in this study were sourced from the MIMIC-IV database, which has been approved by the Institutional Review Board and does not require additional ethical approval for secondary analysis due to its de-identified nature.

### Descriptive statistics

In the descriptive statistics of this study, continuous variables were expressed as median and interquartile range [Median (IQR)]; categorical variables were presented as frequencies and percentages [n(%)]. Missing data were listed separately (Unknown). For between-group comparisons, the following methods were employed: Wilcoxon rank-sum test for continuous variables, and either Pearson’s chi-square test (for large sample groups) or Fisher’s exact test (for small sample groups) for categorical variables, depending on expected frequencies. To control for Type I errors due to multiple comparisons, all statistical test results were adjusted using the False Discovery Rate (FDR) method, yielding corrected q-values. All statistical tests were two-sided, with corrected q-values <0.05 considered statistically significant. Survival analysis will be used to verify whether there is a significant difference in 28-day mortality risk between AKI patients with and without liver cirrhosis in the current dataset.

### Data Preprocessing

This study implemented a systematic data preprocessing workflow to optimize modeling performance. Initially, 28-day mortality was isolated as the target variable, and stratified random sampling was employed to divide the dataset into training and testing sets in an 8:2 ratio, maintaining consistent target variable distribution across both sets. All feature variables underwent min-max normalization, linearly transforming feature values to the [0,1] interval to eliminate the impact of different measurement scales on the model. Feature selection was performed using Recursive Feature Elimination (RFE) based on logistic regression, which evaluated feature importance through an iterative process, ultimately retaining the 15 most contributory features to the prediction outcome. During the feature selection process, five-fold cross-validation was employed to ensure the stability and reliability of the selection results. The training and testing sets were then reconstructed based on the selected feature subset for subsequent model development and validation.

### Model training and validation

This study developed multiple machine learning models for prediction, including Logistic Regression, Linear Discriminant Analysis, Random Forest, XGBoost, LightGBM, and Naive Bayes. The Optuna framework was employed for automatic hyperparameter optimization, with the optimization objective set as the mean accuracy of five-fold cross-validation. For ensemble learning models, optimized parameters included learning rate, tree depth, minimum samples per leaf node, and other key parameters; for linear models, parameters such as regularization strength were optimized. Model evaluation employed a multi-dimensional metric system, including Accuracy, Precision, Recall, and F1 score, while Receiver Operating Characteristic (ROC) curves and Calibration Curves were plotted to assess model discrimination and calibration. To validate model generalizability, performance evaluations were conducted on both training and testing sets, with graphical representations provided for intuitive comparison of predictive performance across models. The model demonstrating optimal predictive performance was ultimately selected as the final prediction tool and saved for subsequent applications.

### Model interpretation

This study employed SHAP (SHapley Additive exPlanations) value analysis to provide in-depth interpretation of the model’s prediction mechanisms. Initially, a model interpreter was constructed using the SHAP framework to analyze feature contributions in the test dataset. Multiple visualization methods were utilized to demonstrate feature importance and impact patterns: Beeswarm plots were used to display the distributed effects of features on prediction outcomes; Heatmaps were employed to present interaction patterns between features; Dependence Plots were utilized to analyze non-linear relationships between important features and prediction results; Force Plots were used to demonstrate the contribution magnitude and direction of features in individual prediction cases; finally, Decision Plots were employed to track the model’s prediction pathway, illustrating the feature contribution trajectory from baseline to final predicted values.

## Results

### Patient characteristics

**Fig 1:**
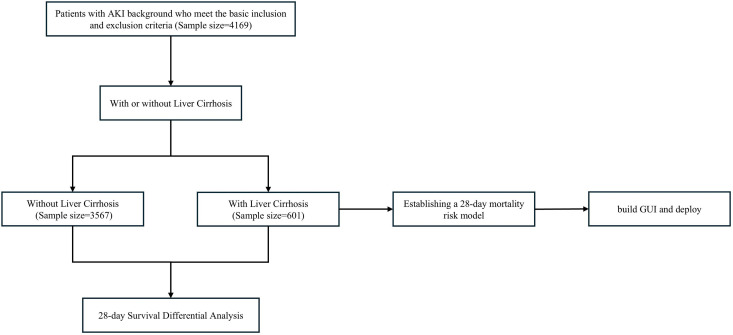
Flow Chart of Patient Selection and Study Design for CHD Mortality Prediction Model.

Among 4,168 AKI patients enrolled, 601 (14%) had concurrent liver cirrhosis. The liver cirrhosis group demonstrated significantly lower age compared to the non-liver cirrhosis group (median age 60 vs 70 years, p < 0.001). While gender distribution showed no significant difference between groups, marital status and insurance type exhibited significant variations (p < 0.001). Laboratory examinations revealed that the liver cirrhosis group had significantly lower platelet counts (94 vs 197 × 10^9/L), hemoglobin (8.82 vs 9.34g/dL), and red blood cell counts than the non-liver cirrhosis group (p < 0.001), with elevated red cell distribution width (18.22 vs 15.68%). Regarding coagulation parameters, the liver cirrhosis group showed significantly higher PT (20.6 vs 15.2s), APTT (46 vs 40s), and INR (1.90 vs 1.38) compared to the non-liver cirrhosis group. Liver function indicators including total bilirubin (5.0 vs 0.8 mg/dL), AST (98 vs 56U/L), and ALT (49 vs 41U/L) were significantly elevated in the liver cirrhosis group. Regarding acid-base balance parameters, the liver cirrhosis group exhibited significantly higher anion gap (16.0 vs 14.9 mmol/L, p < 0.001), lower arterial pH (7.36 vs 7.37, p < 0.001), lower PaCO₂ (39 vs 40 mmHg, p < 0.001), higher lactate levels (2.59 vs 1.94 mmol/L, p < 0.001), and lower total CO₂ (23.0 vs 24.0 mmol/L, p < 0.001) compared to the non-liver cirrhosis group. The distribution of underlying diseases showed that the liver cirrhosis group had significantly lower rates of heart failure (23% vs 47%), myocardial infarction (7.7% vs 19%), and chronic kidney disease (20% vs 33%) compared to the non-liver cirrhosis group, but higher rates of hepatitis (35% vs 3.2%) and sepsis (27% vs 18%). Disease severity scores indicated that the liver cirrhosis group had significantly higher SOFA scores (11 vs 8 points) and APSIII scores (72 vs 59 points) than the non-liver cirrhosis group. These data indicate that AKI patients with liver cirrhosis present distinct demographic characteristics, laboratory parameter alterations, and underlying disease distributions, along with generally higher disease severity scores (Fig 1; S1 Table).

### Survival analysis

**Fig 2:**
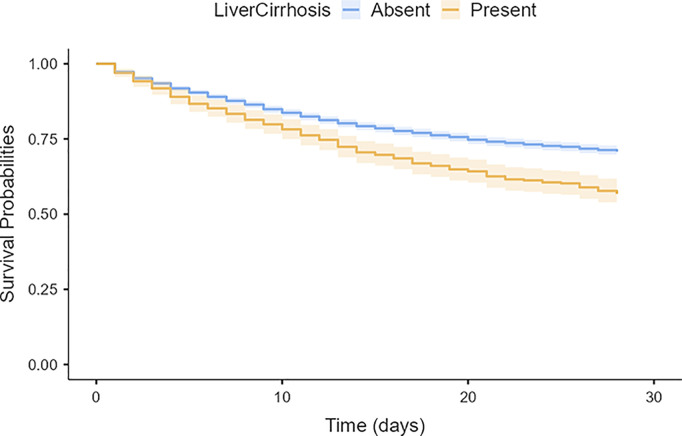
Kaplan-Meier Survival Curves Stratified by Liver Cirrhosis Status in AKI Patients.

Kaplan-Meier survival analysis demonstrated that AKI patients with liver cirrhosis exhibited significantly lower 28-day survival probability compared to those without liver cirrhosis (Log-rank test, χ2 = 46.5, p < 0.001). The survival curves of the two groups began to diverge in the early observation period, with the survival probability gap progressively widening over time. The separation trend in survival curves persisted throughout the observation period without intersection. Analysis of curve slopes indicated that the liver cirrhosis group maintained a higher cumulative mortality risk rate throughout the entire observation period. These data establish liver cirrhosis as a crucial factor influencing AKI patient outcomes, with its impact manifesting from the early stages of the disease (Table 1; Fig 2).

**Table 1:** Log-rank Test Results for 28-day Survival Between Liver Cirrhosis and Non-Liver Cirrhosis Groups.

Test	χ²	df	p
Log-rank	46.5	1	< .001

### Feature selection

Feature selection analysis revealed that the coagulation parameter PT exhibited the highest feature importance, followed by blood gas analysis indicators TotalCO2 and anion gap. The acid-base balance indicator pH and coagulation parameter INR ranked fourth and fifth, respectively. Among blood pressure indicators, systolic pressure NBPS demonstrated higher importance than mean arterial pressure NBPM. Blood lactate and oxygen saturation SpO2 ranked seventh and eighth. Platelet count and APTT, as crucial coagulation function indicators, occupied the ninth and tenth positions. Among basic vital signs and demographic characteristics, white blood cell count, age, heart rate, and respiratory rate showed relatively lower feature importance but were included in the final feature set. These selected features encompassed multiple clinical assessment dimensions including coagulation function, blood gas analysis, organ function, and basic vital signs (S1 Fig).

### Model training

**Fig 3:**
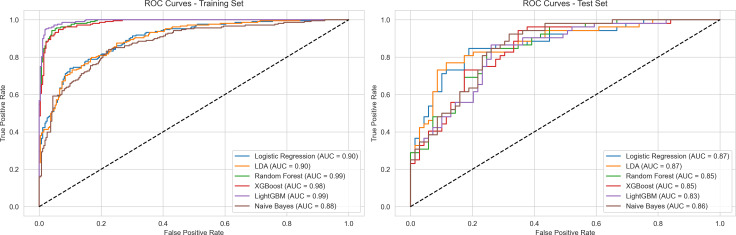
ROC Curves Comparison of Machine Learning Models.

**Fig 4:**
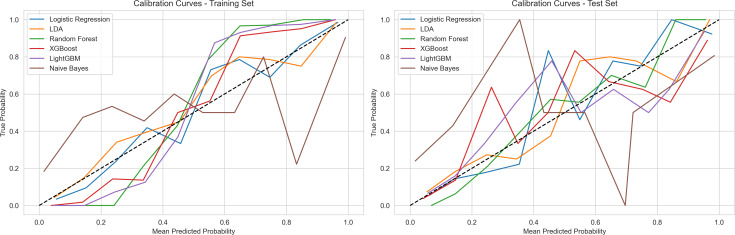
Calibration Performance of Machine Learning Models.

**Fig 5:**
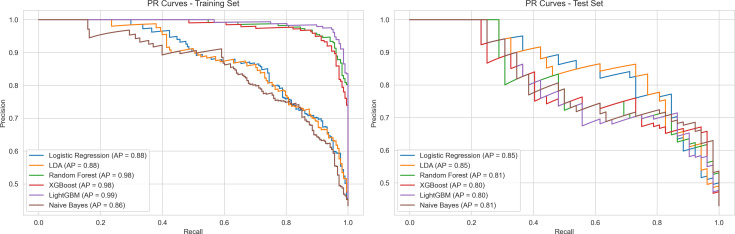
Precision-Recall Analysis of Machine Learning Models.

Model performance evaluation incorporated comprehensive analysis across three dimensions: ROC curves, calibration curves, and PR curves. In the training set, all models demonstrated excellent performance metrics, with LightGBM and XGBoost achieving AUC values of 0.99, followed closely by Random Forest at 0.99, while Logistic Regression and LDA reached 0.90. In the test set, model performance showed anticipated decline but maintained high levels, with the Random Forest model exhibiting the most stable generalization performance, achieving an AUC of 0.85 and an AP value of 0.81 on the PR curve. Calibration curves indicated that Random Forest demonstrated optimal calibration in probability predictions, with curve trends closest to the diagonal line. In contrast, other models such as LightGBM (AUC = 0.83) and Naive Bayes (AUC = 0.86) exhibited greater performance fluctuations in the test set. Through comparison of training and test set performance, considering discriminative ability, probability calibration, and precision-recall trade-off, the Random Forest model demonstrated superior overall performance (Fig 3-5).

### SHAP analysis

**Fig 6:**
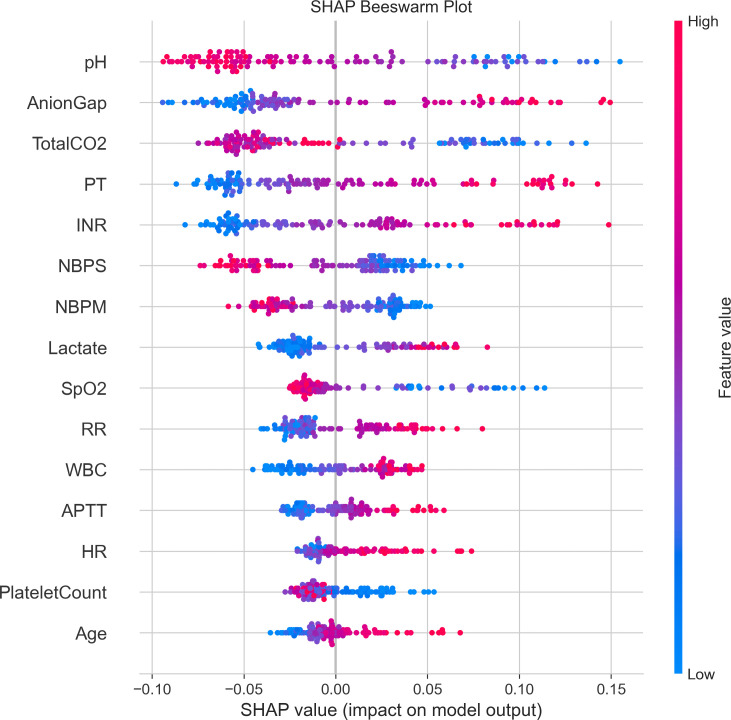
SHAP Values Distribution of Clinical Features.

SHAP analysis revealed the contribution magnitude and dynamic characteristics of each feature to model predictions. In feature importance ranking, pH, anion gap (AnionGap), and total carbon dioxide (TotalCO2) ranked as the top three, with SHAP value distributions ranging from −0.10 to 0.15. Coagulation parameters PT and INR demonstrated significant predictive contributions, exhibiting strong positive influences in high-value ranges. Blood pressure indicators NBPS and NBPM showed moderate predictive contributions, with NBPS displaying relatively higher impact. Lactate and SpO2 exhibited concentrated SHAP value distributions, indicating stable predictive effects within specific ranges. The heatmap further illustrated interaction patterns among features, where pH, anion gap, and TotalCO2 demonstrated pronounced interaction effects across different samples, manifested as strong red-blue contrasts in the heatmap. Temporal analysis indicated that the influence intensity of these key features maintained relative stability throughout the observation period, though significant fluctuations occurred at certain time points (Fig 6,S2 Fig).

SHAP dependence plots uncovered nonlinear relationships between key features and prediction outcomes. Anion gap exhibited significant positive influence in ranges above 0.4, with SHAP values reaching 0.15, and demonstrated notable interaction effects with lactate levels. pH values showed a turning point in the 0.5–0.6 range, with SHAP values displaying negative correlation above this range and positive correlation below, reaching maximum contributions of ±0.15. TotalCO2 demonstrated positive influence in regions below 0.4, with SHAP values of 0.12, transitioning to negative influence beyond 0.4. INR and PT exhibited similar influence patterns, both showing strong positive predictive effects in ranges above 0.4, with maximum SHAP values of 0.15, and minimal interaction effects with other indicators. NBPM and NBPS displayed similar influence patterns, both showing inflection points around 0.4, with SHAP value ranges of −0.06 to 0.04 for NBPM and −0.08 to 0.06 for NBPS, both exhibiting negative influences in high-value ranges. These nonlinear relationships and interaction effects highlight the complex physiological and pathological mechanisms captured by the prediction model (S3-S9 Fig)

SHAP force plots demonstrated feature contribution distributions across five randomly selected case samples from the dataset. These samples exhibited baseline prediction values of 0.47, 0.88, 0.25, 0.37, and 0.33, reflecting varying risk prediction levels across different cases. Regarding feature contributions, blood pressure indicators (NBPS and NBPM) demonstrated significant influence across multiple samples, with NBPM (0.2933) and NBPS (0.2993) showing positive contributions in the sample with a baseline value of 0.47. Among blood gas analysis indicators, pH and TotalCO2 exhibited opposing directional influences across multiple samples, notably in the sample with a baseline value of 0.25, where TotalCO2 (−0.5463) demonstrated strong negative effects. Coagulation parameters showed prominence in high-risk samples (baseline value 0.88), with INR (0.3305) and PT (0.3406) both demonstrating significant positive influences. Vital sign indicators including HR and RR showed marked variation in influence across different samples, exemplified by HR (0.6188) exhibiting the most significant positive contribution in the sample with a baseline value of 0.47 (S9-S14 Fig).

### Decision curve analysis

**Fig 7:**
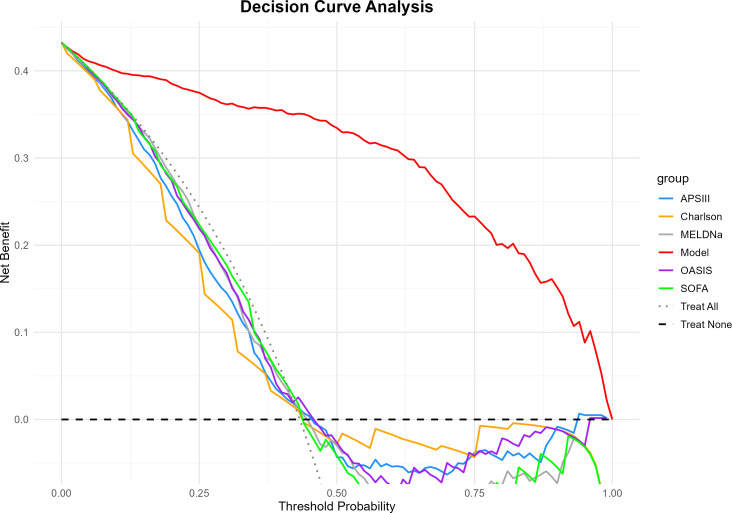
Comparative Decision Curve Analysis of Machine Learning Model versus Traditional Critical Care Scoring Systems for Clinical Benefit Assessment.

Decision curve analysis (DCA) results demonstrated superior clinical decision-making value of the constructed machine learning model compared to traditional scoring systems. Across the threshold probability range of 0–1, the model (red line) consistently exhibited higher net benefit than five traditional scoring systems: APSIII, Charlson, MELDNa, OASIS, and SOFA. In the low-risk threshold interval (0–0.25), all scoring methods showed comparable performance with net benefit values decreasing from 0.42 to 0.30. In the moderate-risk threshold interval (0.25–0.75), the model maintained substantially higher net benefit (0.35–0.20), while traditional scoring systems converged toward 0, with Charlson showing the lowest performance among the five systems. MELDNa (grey line) demonstrated performance comparable to other clinical scores (APSIII, OASIS, and SOFA), following a similar trajectory but with slightly lower net benefit in some portions of the moderate-risk threshold range. In the high-risk threshold interval (0.75–1.0), the model’s net benefit gradually decreased from 0.20 to 0, maintaining superiority over traditional systems which exhibited minimal fluctuations near the zero line. The “Treat All” strategy demonstrated a linear decrease in net benefit with increasing threshold probability, intersecting with other curves at approximately 0.50 threshold, while the “Treat None” strategy maintained constant net benefit at 0. These two baseline curves further validated the model’s enhanced discriminative capability in clinical decision support (Fig 7).

## Discussion

This study developed a prediction model for 28-day mortality risk in AKI patients based on the MIMIC-IV database. Results indicated that AKI patients with liver cirrhosis exhibited distinct clinical characteristics and elevated mortality risk. Survival analysis confirmed significantly lower 28-day survival rates in the liver cirrhosis group compared to the non-liver cirrhosis group. In model development, recursive feature elimination identified key predictive factors spanning multiple dimensions, including coagulation function, blood gas analysis, and basic vital signs. pH, anion gap, and TotalCO2 contributed most substantially to prediction outcomes, with significant interaction effects observed among these indicators. Among various machine learning algorithms compared, the random forest model demonstrated superior generalization performance, achieving an AUC of 0.85 and precision-recall area under curve of 0.81. Decision curve analysis further confirmed the model’s superior clinical decision-making value compared to traditional scoring systems, particularly excelling in moderate-risk threshold intervals.

Acute kidney injury (AKI) in the context of liver cirrhosis presents a significant clinical challenge, particularly due to the complex interplay between renal and hepatic dysfunction. The definition of AKI in patients with cirrhosis has evolved, with the International Club of Ascites (IAC) proposing that AKI is characterized by an increase in serum creatinine (SCr) of 0.3 mg/dl within 48 hours or a 50% increase from baseline within 7 days. This definition underscores the urgency of early detection, as timely intervention can potentially reverse kidney failure and improve survival rates [8]. The prevalence of AKI among hospitalized patients with liver cirrhosis ranges from 20% to 50%, indicating a substantial burden on this patient population [5,9,10]. The pathophysiology of AKI in cirrhosis is multifactorial, with hepatorenal syndrome (HRS) being a critical component. HRS is characterized by functional renal failure due to severe intrarenal vasoconstriction, often triggered by splanchnic arterial vasodilation and circulatory dysfunction, particularly in patients with ascites [11,12]. Type 1 HRS represents the most severe form of AKI in cirrhosis, typically progressing rapidly and associated with poor outcomes [11]. The renal impairment observed in these patients is not merely a consequence of liver dysfunction but is also exacerbated by factors such as diuretic use, gastrointestinal bleeding, and infections, which can precipitate or worsen AKI [10,13].The intersection of AKI and liver cirrhosis represents a significant clinical challenge, characterized by high morbidity and poor outcomes. In this context, the definition and understanding of AKI continue to evolve, emphasizing the necessity of vigilant monitoring and early intervention. Therefore, the machine learning model developed in this study holds substantial clinical application value in this field, achieving accurate outcome prediction for these high-risk patients through the integration of multidimensional clinical indicators, providing clinicians with an objective and reliable risk assessment tool.

In our further SHAP interpretation of the model, pH levels demonstrated substantial influence on short-term mortality risk. Existing research indicates that low serum bicarbonate levels, indicating metabolic acidosis, correlate with increased mortality in patients with liver cirrhosis and AKI [14,15]. This relationship suggests that maintaining more neutral pH values contributes to improved outcomes. Additionally, the presence of acidosis leads to alterations in drug metabolism and excretion, complicating the management of liver cirrhosis and AKI [16]. Furthermore, pH affects systemic inflammatory responses, which typically intensify in patients with liver cirrhosis and AKI. Elevated inflammatory cytokine levels, including interleukin-6 and tumor necrosis factor-α, correlate with adverse outcomes in this population [17,18]. Acid-base status modulates these inflammatory pathways, potentially leading to a vicious cycle where acidosis exacerbates inflammation, further compromising renal function and worsening liver disease [19]. AG represents a crucial measurement for assessing acid-base balance, with particular utility in liver cirrhosis. Elevated AG indicates the presence of unmeasured anions, such as lactate, which accumulate during septic episodes or tissue hypoperfusion, both common in cirrhotic patients experiencing AKI. Studies demonstrate that higher AG values correlate with increased in-hospital mortality and poor prognosis in sepsis-associated AKI patients, emphasizing the relationship between AG and outcomes in this population [20,21]. Moreover, AG interpretation in cirrhotic patients requires consideration of unique pathophysiological changes associated with liver disease. For instance, creatinine synthesis impairment in liver cirrhosis leads to potentially misleading serum creatinine levels, preventing accurate reflection of renal function [22,23]. This complicates AKI assessment, as traditional markers fail to provide reliable indicators of renal function impairment. Consequently, AG serves as an auxiliary marker, providing additional information to support clinical decision-making [23,24]. TotalCO2, as an indicator of acid-base metabolic balance reflecting blood bicarbonate concentration, indicates metabolic disorders in patients with liver cirrhosis and AKI. In cirrhotic patients, particularly those with decompensated liver disease, acid-base alterations occur frequently due to impaired hepatic function and renal clearance capacity [25,26]. Elevated TotalCO2 levels indicate metabolic alkalosis, occurring with diuretic use or volume depletion, both prevalent in cirrhotic patients [10]. Conversely, low TotalCO2 levels indicate metabolic acidosis, potentially resulting from lactate accumulation due to hepatic dysfunction or renal failure [27,28]. The prognostic impact of TotalCO2 values in liver cirrhosis and AKI patients is emphasized through their association with clinical outcomes. Research demonstrates that patients with lower TotalCO2 levels experience poorer prognoses, including higher mortality rates [5]. This relationship attributes to the underlying pathophysiology of liver disease and renal impairment, where inability to maintain acid-base homeostasis reflects disease severity. Furthermore, TotalCO2 levels influence the interpretation of other laboratory values, including creatinine, a key marker for assessing renal function in cirrhotic patients [29]. For patients with AKI and liver cirrhosis, acid-base metabolism-related indicators represent crucial factors influencing short-term mortality risk, providing important guidance for treatment strategy development. Targeted correction of acid-base imbalances emerges as a key therapeutic component for improving outcomes in these patients. Beyond the established predictive value of pH, anion gap, and total CO2, our findings highlight the significant roles of PaCO2 and lactate levels in mortality prediction for AKI patients with liver cirrhosis. The observed lower PaCO2 levels (39 vs 40 mmHg, p < 0.001) in the cirrhosis group likely reflect compensatory respiratory alkalosis in response to metabolic acidosis, a physiological attempt to normalize pH that indicates systemic stress. This respiratory compensation mechanism, when insufficient to normalize acid-base balance, signals severe metabolic derangement and correlates with poorer outcomes. Similarly, elevated lactate levels (2.59 vs 1.94 mmol/L, p < 0.001) in cirrhotic patients represent a critical prognostic marker through multiple pathophysiological mechanisms: impaired hepatic lactate clearance due to compromised liver function, tissue hypoperfusion from hemodynamic instability, and microcirculatory dysfunction exacerbated by systemic inflammation. The interaction between these parameters creates a complex acid-base profile where lactate accumulation drives metabolic acidosis, triggering respiratory compensation reflected in decreased PaCO2, while simultaneously contributing to organ dysfunction through tissue hypoxia and cellular energetic failure. Early identification and targeted correction of these specific acid-base disturbances, particularly lactate-driven acidosis, may provide a crucial therapeutic window for intervention before irreversible organ damage occurs.

This study has several limitations. The MIMIC database, while providing large-scale clinical data, originates from a single medical center within the U.S. healthcare system, creating inherent constraints in extrapolating the research findings to diverse healthcare environments and populations worldwide. Additionally, our methodological framework exclusively utilized clinical data from the initial 24-hour admission period, omitting the temporal evolution of patient parameters throughout hospitalization. The deliberate exclusion of treatment-related variables, implemented to minimize confounding effects, enhanced early predictive capabilities but simultaneously neglected the therapeutic interventions’ influence on patient outcomes. Furthermore, we were unable to accurately identify and analyze ACLF triggers or distinguish HRS-AKI from other types of AKI due to the inherent limitations of retrospective data from the MIMIC-IV database, which lacks standardized documentation for these specific clinical entities. Finally, the developed prediction model currently lacks prospective external validation, necessitating additional practical verification to establish the model’s clinical utility before widespread implementation.

## Conclusion

The machine learning prediction model developed in this study based on the MIMIC-IV database demonstrated excellent performance in assessing 28-day mortality risk for AKI patients with liver cirrhosis. The model achieved an AUC of 0.85 through the integration of key clinical indicators including pH, AG, and TotalCO2. SHAP analysis revealed the central role of acid-base metabolic balance in prognostic assessment, while decision curve analysis confirmed the model’s superior clinical utility compared to traditional scoring systems. This predictive tool provides clinicians with objective risk quantification basis, holding significant importance for optimizing treatment strategies and improving patient outcomes.

## Supporting information

S1 Fig Feature Coefficients of Selected Clinical Variables in CHD Mortality Prediction Model. (TIFF)

S2 Fig Feature Contribution Patterns Across Individual Cases in Prediction Model. (TIFF)

S3 Fig Nonlinear Distribution Pattern of pH Values and Its Interaction Effects on Outcomes. (TIFF)

S4 Fig Nonlinear Distribution Pattern of Anion Gap and Its Interaction Effects on Outcomes. (TIFF)

S5 Fig Nonlinear Distribution Pattern of Total CO2 and Its Interaction Effects on Outcomes. (TIFF)

S6 Fig Nonlinear Distribution Pattern of PT and Its Interaction Effects on Outcomes. (TIFF)

S7 Fig Nonlinear Distribution Pattern of INR and Its Interaction Effects on Outcomes. (TIFF)

S8 Fig Nonlinear Distribution Pattern of NBPS and Its Interaction Effects on Outcomes. (TIFF)

S9 Fig Nonlinear Distribution Pattern of NBPM and Its Interaction Effects on Outcomes. (TIFF)

S10 Fig Force Plot Analysis with Dominant HR (0.6188) and RR (0.5472) Effects at Base Value 0.47. (TIFF)

S11 Fig Force Plot Analysis with Critical INR (0.3305) and PT (0.3406) Impact at Base Value 0.88. (TIFF)

S12 Fig Force Plot Analysis with Prominent HR (0.7102) and RR (0.6327) Contributions at Base Value 0.25. (TIFF)

S13 Fig Force Plot Analysis with Significant AnionGap (0.3614) and TotalCO2 (0.3668) Influence at Base Value 0.37. (TIFF)

S14 Fig Force Plot Analysis with Notable SpO2 (0.3599) and NBPS (0.2493) Features at Base Value 0.33. (TIFF)

S1 Table Patients Baseline. (DOCX)
