# Supplementary figures and images for: Machine learning-based prediction model for 28-day mortality in acute kidney injury patients with liver cirrhosis: A MIMIC-IV database analysis
Source: PLoS One. 2025 Sep 8;20(9):e0328662. doi: 10.1371/journal.pone.0328662 (PMC12416639; doi:10.1371/journal.pone.0328662)

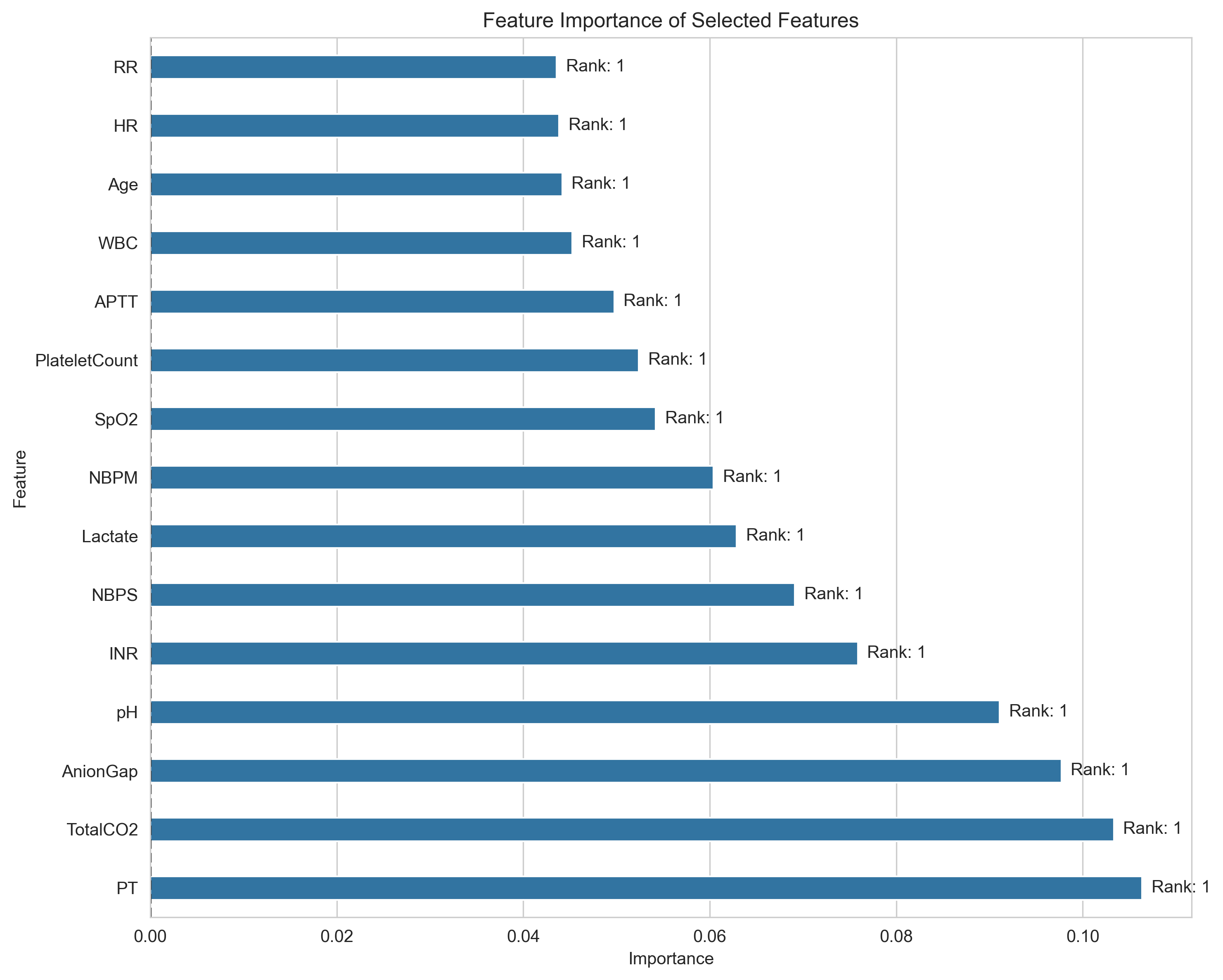

Supplement: S1 Fig — (TIFF) [file pone.0328662.s001.tiff]

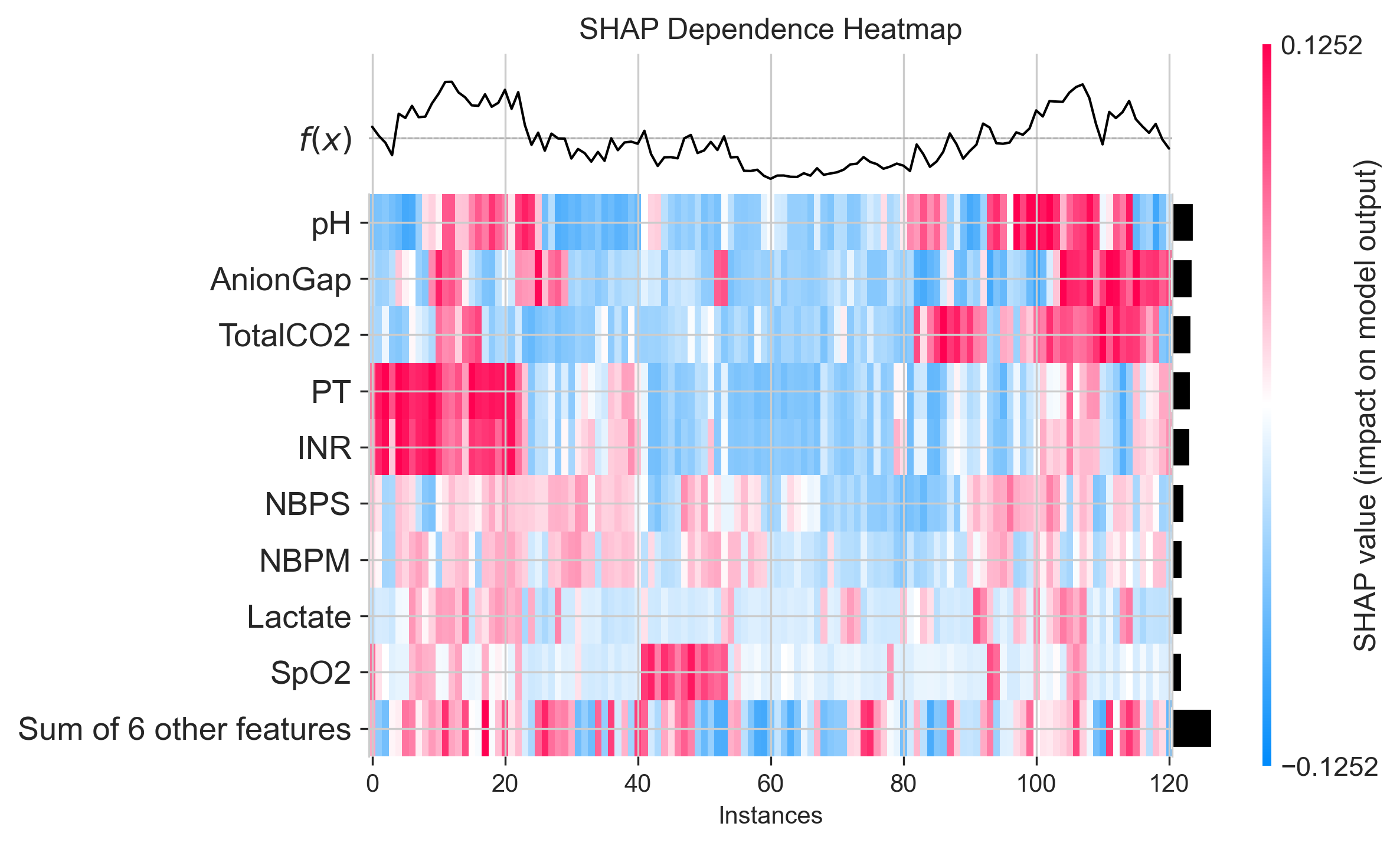

Supplement: S2 Fig — (TIFF) [file pone.0328662.s002.tiff]

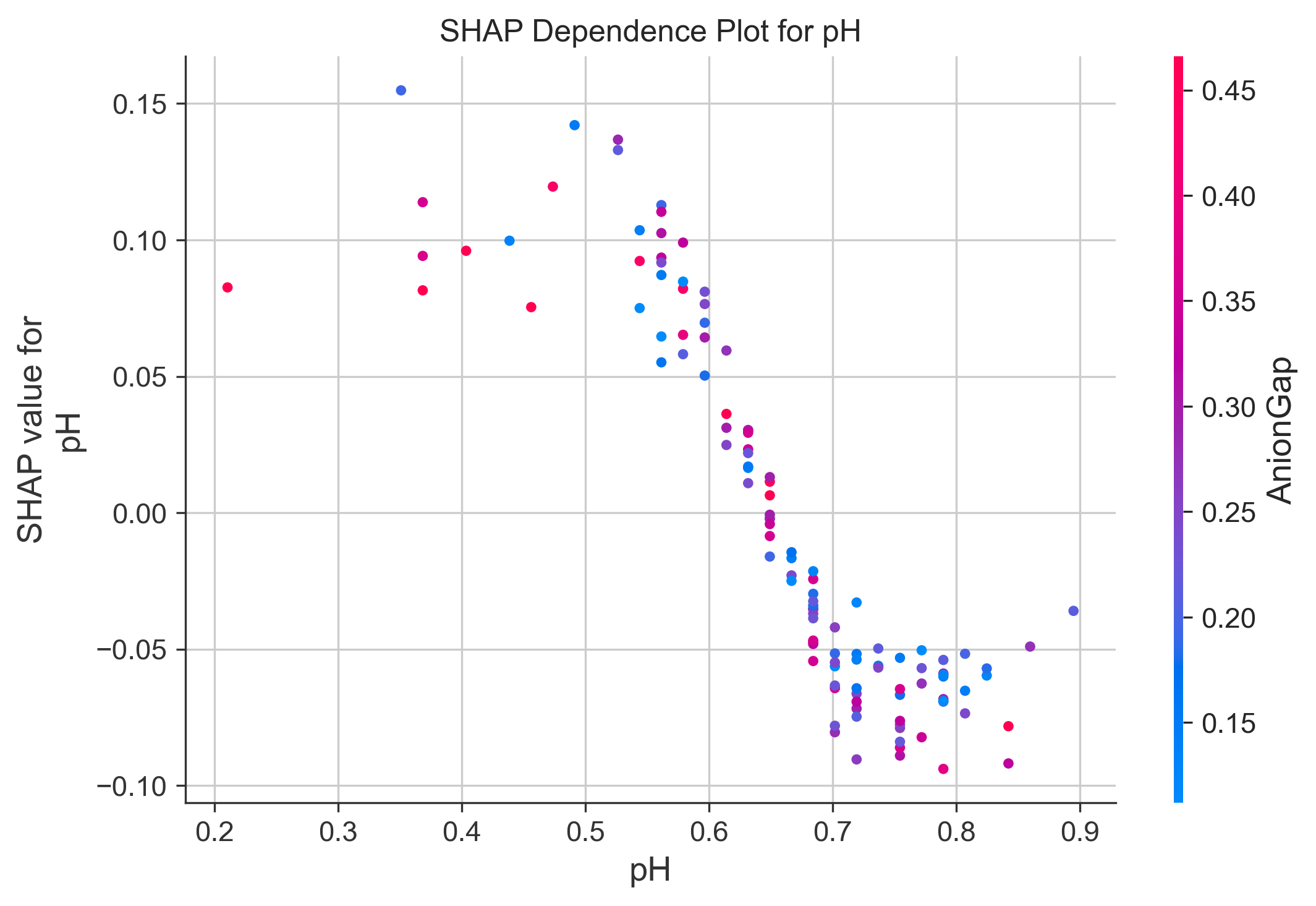

Supplement: S3 Fig — (TIFF) [file pone.0328662.s003.tiff]

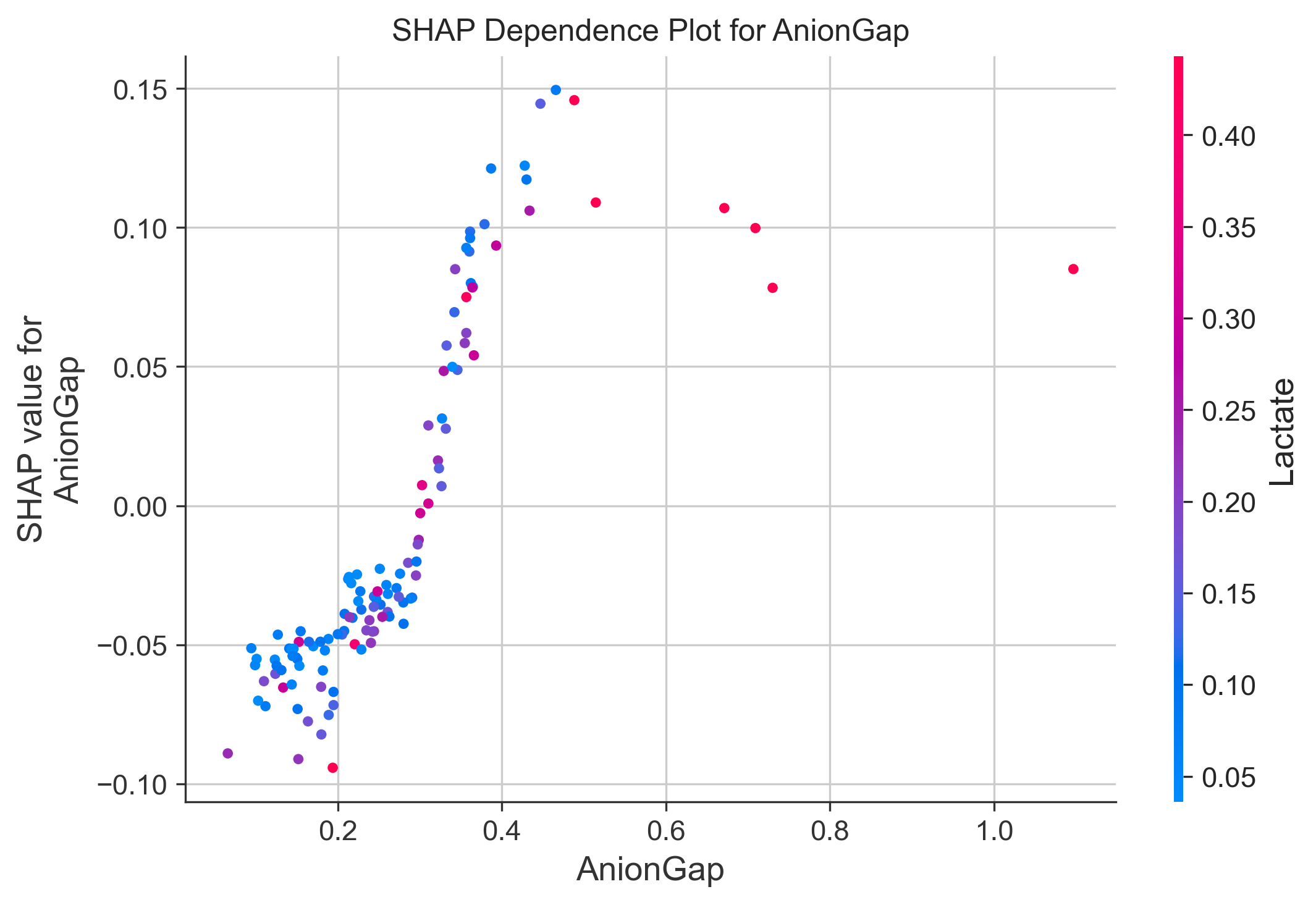

Supplement: S4 Fig — (TIFF) [file pone.0328662.s004.tiff]

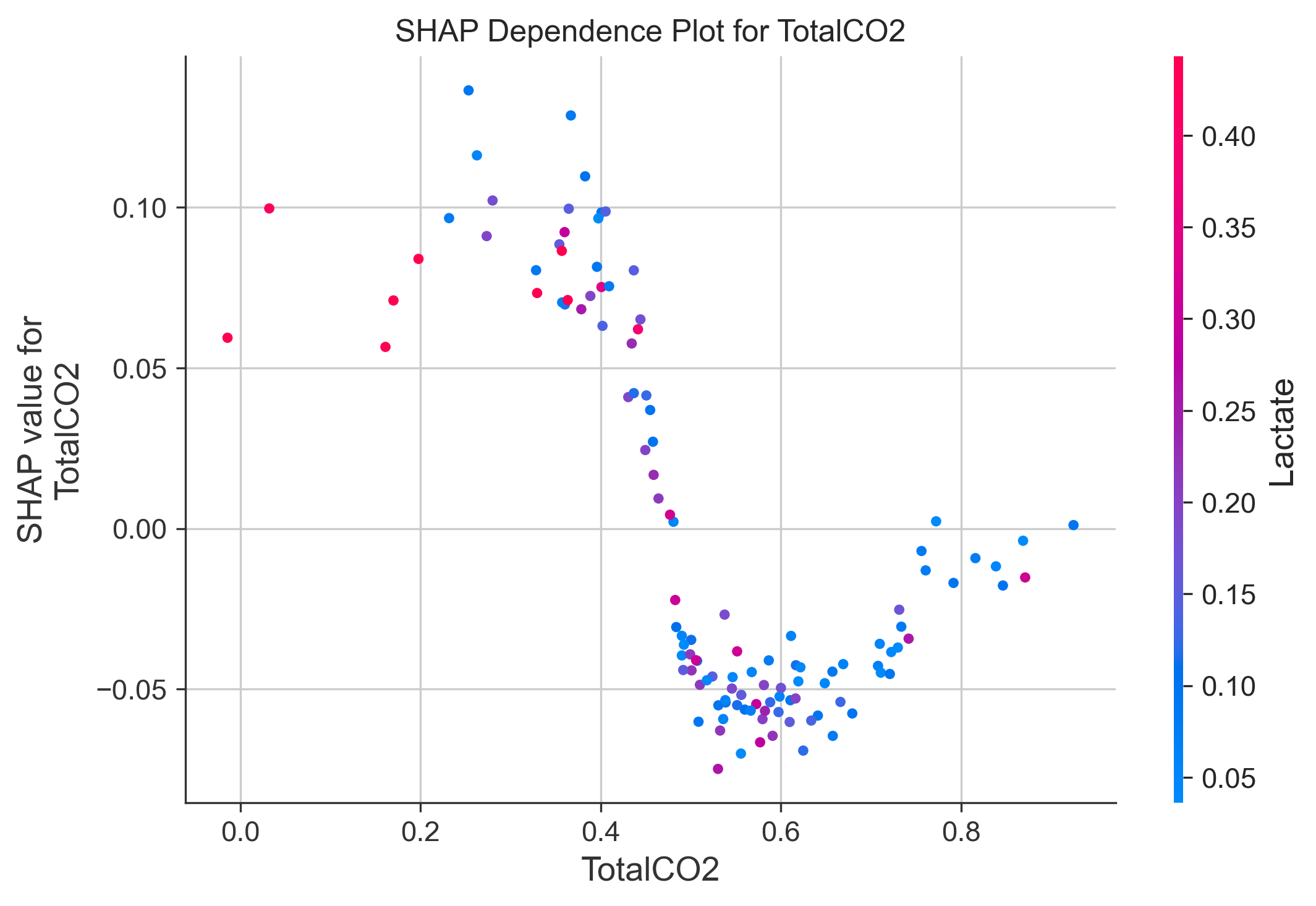

Supplement: S5 Fig — (TIFF) [file pone.0328662.s005.tiff]

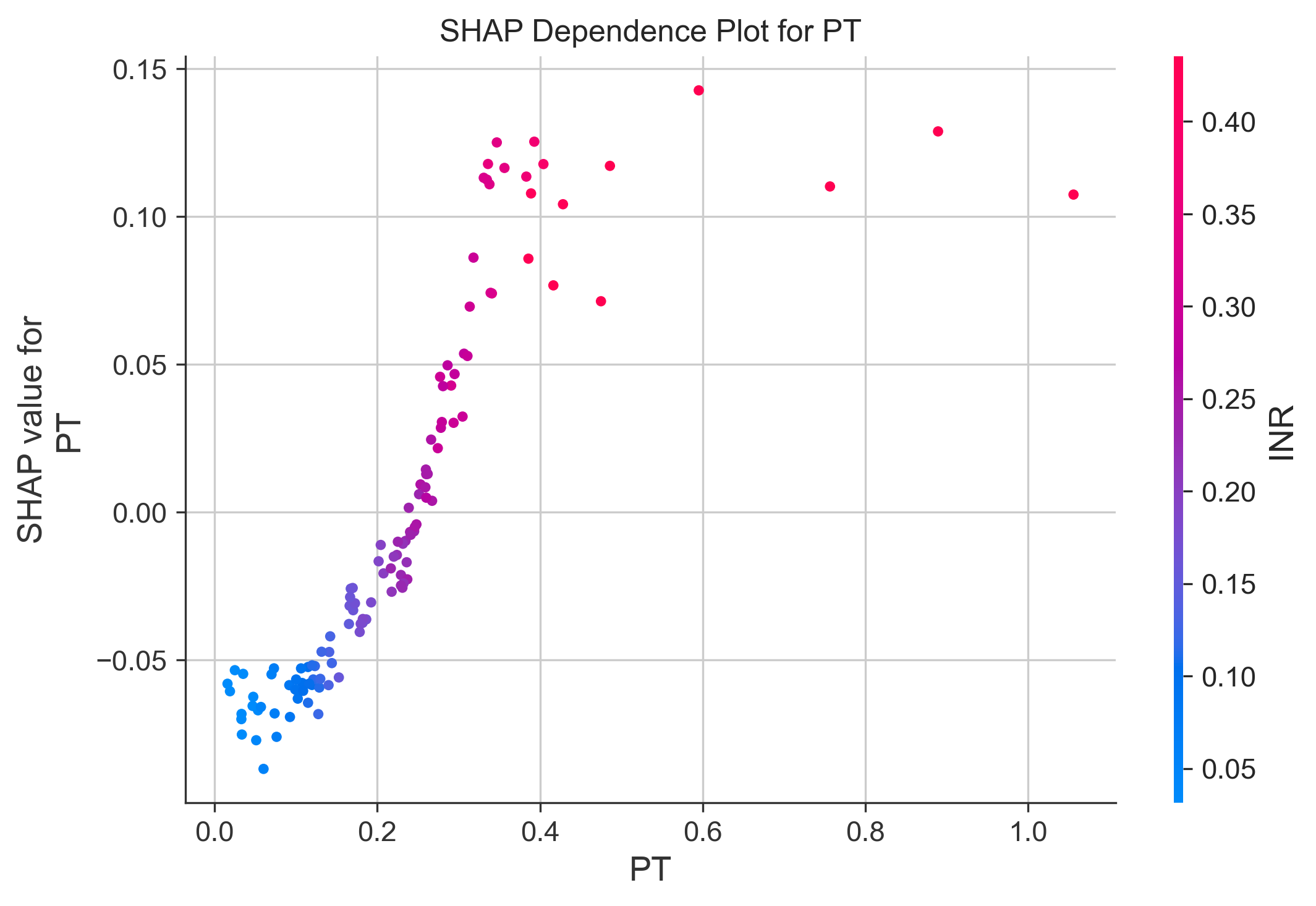

Supplement: S6 Fig — (TIFF) [file pone.0328662.s006.tiff]

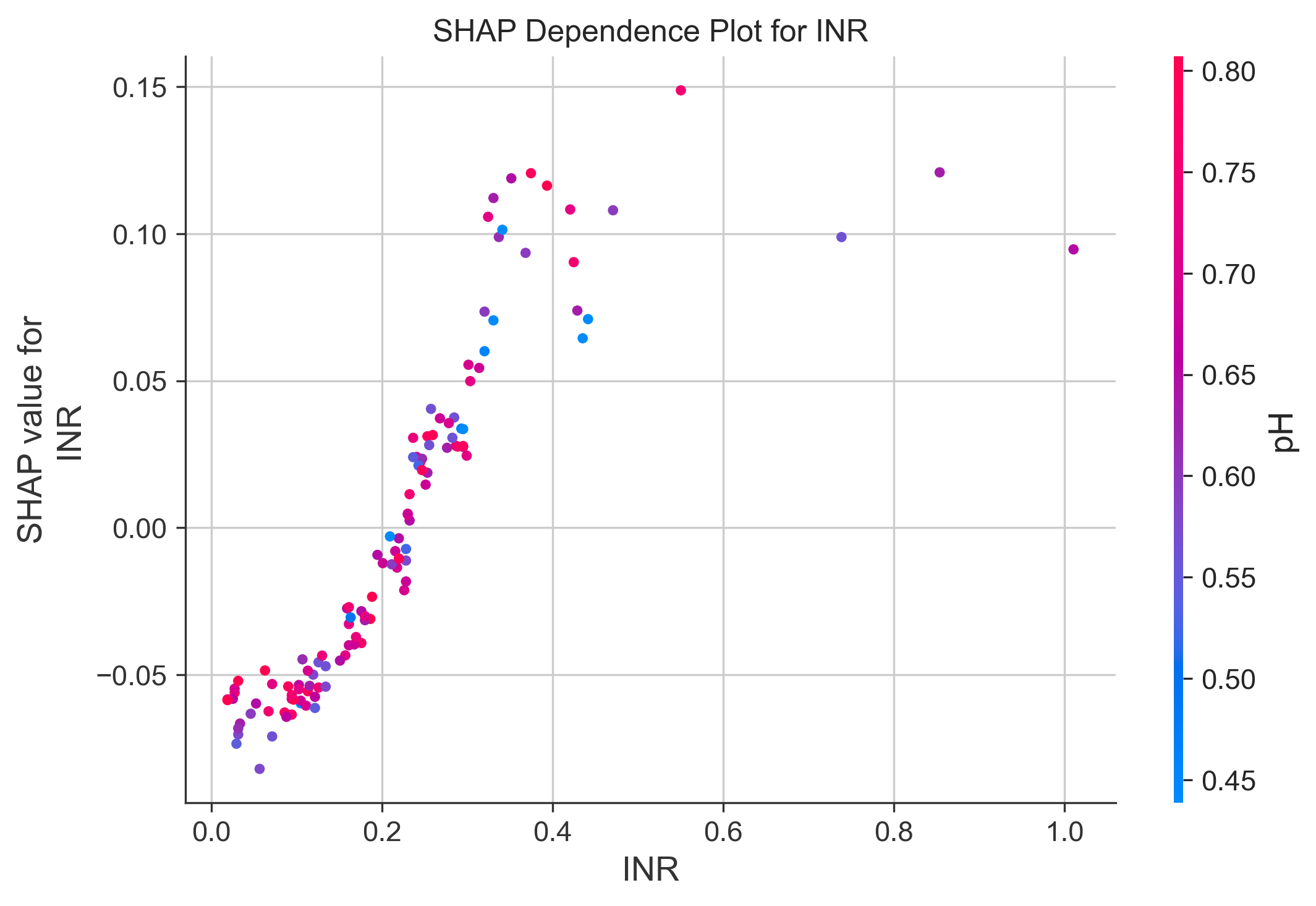

Supplement: S7 Fig — (TIFF) [file pone.0328662.s007.tiff]

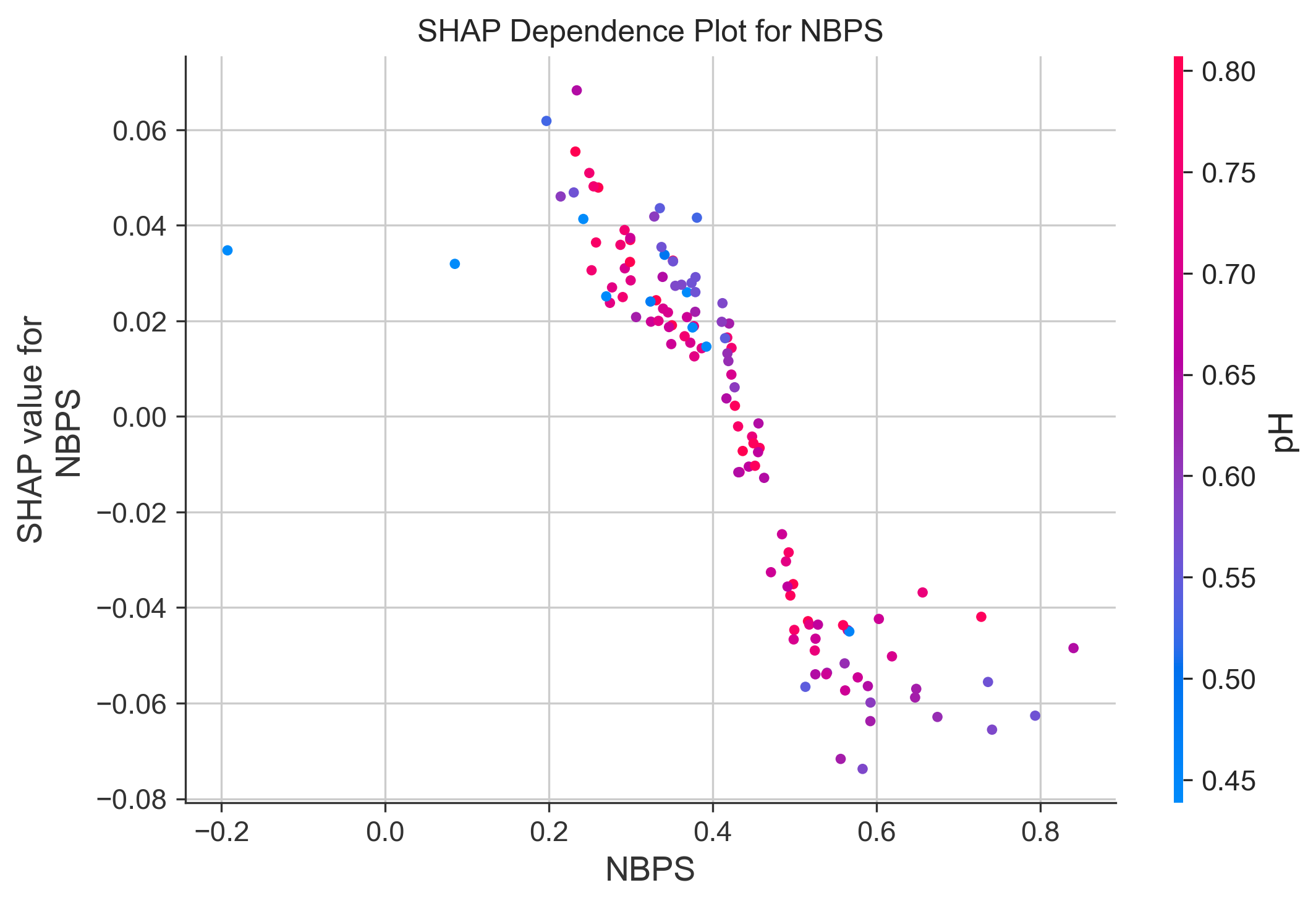

Supplement: S8 Fig — (TIFF) [file pone.0328662.s008.tiff]

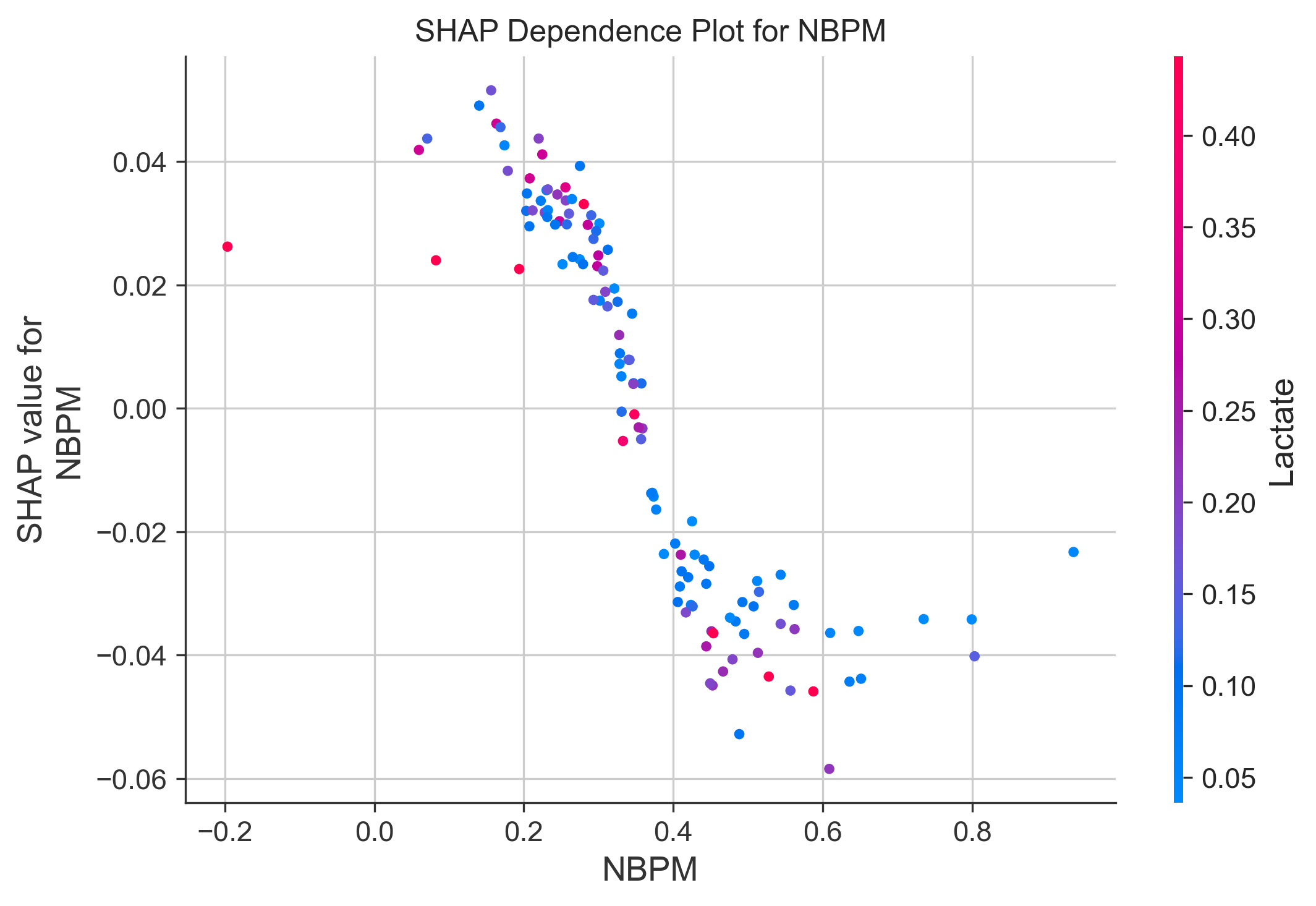

Supplement: S9 Fig — (TIFF) [file pone.0328662.s009.tiff]

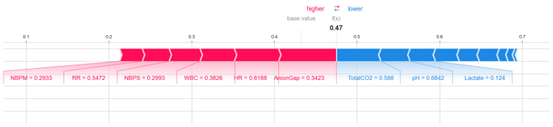

Supplement: S10 Fig — (TIFF) [file pone.0328662.s010.tiff]

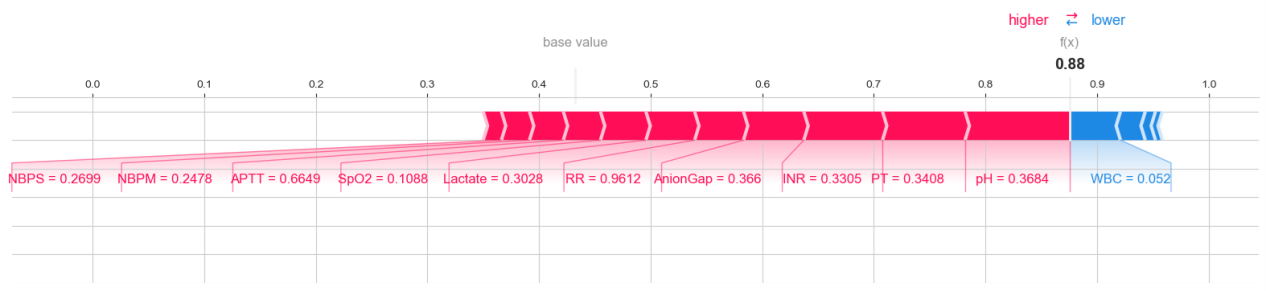

Supplement: S11 Fig — (TIFF) [file pone.0328662.s011.tiff]

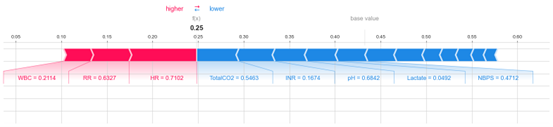

Supplement: S12 Fig — (TIFF) [file pone.0328662.s012.tiff]

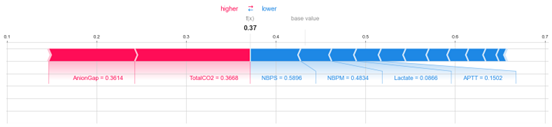

Supplement: S13 Fig — (TIFF) [file pone.0328662.s013.tiff]

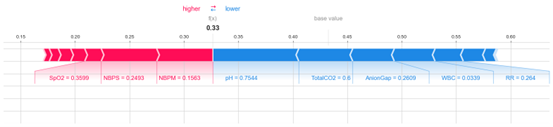

Supplement: S14 Fig — (TIFF) [file pone.0328662.s014.tiff]
