# Supplementary material for: Machine learning-based prediction model for 28-day mortality in acute kidney injury patients with liver cirrhosis: A MIMIC-IV database analysis
Source: PLoS One. 2025 Sep 8;20(9):e0328662. doi: 10.1371/journal.pone.0328662 (PMC12416639; doi:10.1371/journal.pone.0328662)
Supplement: S1 Table — (DOCX) [file pone.0328662.s015.docx]

| **Characteristic** | **N** | **Liver Cirrhosis** | | | **p-value** | **q-value** |
| --- | --- | --- | --- | --- | --- | --- |
|  |  | **Absent**  **N = 3567 (86%)** | | **Present**  **N = 601 (14%)** |  |  |
| Age | 4,168 | 70 (60, 80) | 60 (52, 68) | | <0.001 | <0.001 |
| Gender | 4,168 |  |  | | 0.39 | 0.44 |
| Female |  | 1,354 (38%) | 217 (36%) | |  |  |
| Male |  | 2,213 (62%) | 384 (64%) | |  |  |
| Race | 4,168 |  |  | |  |  |
| AMERICAN INDIAN/ALASKA NATIVE |  | 7 (0.2%) | 1 (0.2%) | |  |  |
| ASIAN |  | 36 (1.0%) | 6 (1.0%) | |  |  |
| ASIAN - ASIAN INDIAN |  | 6 (0.2%) | 2 (0.3%) | |  |  |
| ASIAN - CHINESE |  | 32 (0.9%) | 4 (0.7%) | |  |  |
| ASIAN - KOREAN |  | 4 (0.1%) | 0 (0%) | |  |  |
| ASIAN - SOUTH EAST ASIAN |  | 11 (0.3%) | 3 (0.5%) | |  |  |
| BLACK/AFRICAN |  | 9 (0.3%) | 0 (0%) | |  |  |
| BLACK/AFRICAN AMERICAN |  | 261 (7.3%) | 35 (5.8%) | |  |  |
| BLACK/CAPE VERDEAN |  | 18 (0.5%) | 2 (0.3%) | |  |  |
| BLACK/CARIBBEAN ISLAND |  | 22 (0.6%) | 5 (0.8%) | |  |  |
| HISPANIC OR LATINO |  | 16 (0.4%) | 2 (0.3%) | |  |  |
| HISPANIC/LATINO - CENTRAL AMERICAN |  | 2 (<0.1%) | 0 (0%) | |  |  |
| HISPANIC/LATINO - COLUMBIAN |  | 2 (<0.1%) | 0 (0%) | |  |  |
| HISPANIC/LATINO - CUBAN |  | 1 (<0.1%) | 1 (0.2%) | |  |  |
| HISPANIC/LATINO - DOMINICAN |  | 32 (0.9%) | 8 (1.3%) | |  |  |
| HISPANIC/LATINO - GUATEMALAN |  | 4 (0.1%) | 0 (0%) | |  |  |
| HISPANIC/LATINO - HONDURAN |  | 2 (<0.1%) | 2 (0.3%) | |  |  |
| HISPANIC/LATINO - MEXICAN |  | 3 (<0.1%) | 2 (0.3%) | |  |  |
| HISPANIC/LATINO - PUERTO RICAN |  | 44 (1.2%) | 18 (3.0%) | |  |  |
| HISPANIC/LATINO - SALVADORAN |  | 2 (<0.1%) | 1 (0.2%) | |  |  |
| MULTIPLE RACE/ETHNICITY |  | 1 (<0.1%) | 0 (0%) | |  |  |
| NATIVE HAWAIIAN OR OTHER PACIFIC ISLANDER |  | 8 (0.2%) | 0 (0%) | |  |  |
| OTHER |  | 113 (3.2%) | 17 (2.8%) | |  |  |
| PATIENT DECLINED TO ANSWER |  | 22 (0.6%) | 4 (0.7%) | |  |  |
| PORTUGUESE |  | 8 (0.2%) | 5 (0.8%) | |  |  |
| SOUTH AMERICAN |  | 1 (<0.1%) | 0 (0%) | |  |  |
| UNABLE TO OBTAIN |  | 59 (1.7%) | 12 (2.0%) | |  |  |
| UNKNOWN |  | 535 (15%) | 86 (14%) | |  |  |
| WHITE |  | 2,209 (62%) | 371 (62%) | |  |  |
| WHITE - BRAZILIAN |  | 4 (0.1%) | 1 (0.2%) | |  |  |
| WHITE - EASTERN EUROPEAN |  | 7 (0.2%) | 0 (0%) | |  |  |
| WHITE - OTHER EUROPEAN |  | 59 (1.7%) | 11 (1.8%) | |  |  |
| WHITE - RUSSIAN |  | 27 (0.8%) | 2 (0.3%) | |  |  |
| Weight | 4,138 | 84 (70, 100) | 85 (73, 103) | | 0.026 | 0.034 |
| Unknown |  | 24 | 6 | |  |  |
| Insurance | 4,168 |  |  | | <0.001 | <0.001 |
| Medicaid |  | 202 (5.7%) | 80 (13%) | |  |  |
| Medicare |  | 1,757 (49%) | 195 (32%) | |  |  |
| Other |  | 1,608 (45%) | 326 (54%) | |  |  |
| Language | 4,168 |  |  | | 0.88 | 0.9 |
| ? |  | 390 (11%) | 67 (11%) | |  |  |
| ENGLISH |  | 3,177 (89%) | 534 (89%) | |  |  |
| MaritalStatus | 3,715 |  |  | | <0.001 | <0.001 |
| DIVORCED |  | 257 (8.1%) | 58 (11%) | |  |  |
| MARRIED |  | 1,627 (51%) | 239 (45%) | |  |  |
| SINGLE |  | 857 (27%) | 204 (38%) | |  |  |
| WIDOWED |  | 441 (14%) | 32 (6.0%) | |  |  |
| Unknown |  | 385 | 68 | |  |  |
| WBC | 4,168 | 12.2 (9.4, 15.5) | 11.2 (8.0, 15.3) | | <0.001 | <0.001 |
| RBC | 4,168 | 3.14 (2.88, 3.49) | 2.81 (2.55, 3.13) | | <0.001 | <0.001 |
| PlateletCount | 4,168 | 197 (136, 270) | 94 (64, 144) | | <0.001 | <0.001 |
| Hemoglobin | 4,168 | 9.34 (8.52, 10.32) | 8.82 (8.11, 9.91) | | <0.001 | <0.001 |
| RDW | 4,167 | 15.68 (14.47, 17.20) | 18.22 (16.51, 20.21) | | <0.001 | <0.001 |
| Unknown |  | 1 | 0 | |  |  |
| Hematocrit | 4,168 | 28.6 (26.3, 31.3) | 26.9 (24.8, 29.4) | | <0.001 | <0.001 |
| Albumin | 3,903 | 2.86 (2.47, 3.30) | 3.00 (2.68, 3.45) | | <0.001 | <0.001 |
| Unknown |  | 258 | 7 | |  |  |
| Sodium | 4,168 | 138.8 (136.2, 141.4) | 137.6 (134.6, 140.8) | | <0.001 | <0.001 |
| Potassium | 4,168 | 4.19 (3.97, 4.42) | 4.11 (3.89, 4.41) | | <0.001 | <0.001 |
| CalciumTotal | 4,167 | 8.33 (7.94, 8.70) | 8.57 (8.14, 9.02) | | <0.001 | <0.001 |
| Unknown |  | 1 | 0 | |  |  |
| Chloride | 4,168 | 102.7 (99.4, 106.0) | 102.4 (98.1, 105.9) | | 0.048 | 0.059 |
| Glucose | 4,168 | 135 (117, 166) | 133 (115, 161) | | 0.028 | 0.036 |
| AnionGap | 4,168 | 14.9 (13.2, 17.2) | 16.0 (13.6, 18.4) | | <0.001 | <0.001 |
| pH | 4,166 | 7.37 (7.33, 7.41) | 7.36 (7.32, 7.40) | | <0.001 | <0.001 |
| Unknown |  | 2 | 0 | |  |  |
| pCO2 | 4,154 | 40 (36, 44) | 39 (35, 43) | | <0.001 | <0.001 |
| Unknown |  | 13 | 1 | |  |  |
| pO2 | 4,154 | 110 (87, 139) | 100 (83, 123) | | <0.001 | <0.001 |
| Unknown |  | 13 | 1 | |  |  |
| Lactate | 4,144 | 1.94 (1.43, 2.86) | 2.59 (1.96, 3.98) | | <0.001 | <0.001 |
| Unknown |  | 23 | 1 | |  |  |
| TotalCO2 | 4,155 | 24.0 (21.3, 26.5) | 23.0 (20.2, 25.6) | | <0.001 | <0.001 |
| Unknown |  | 12 | 1 | |  |  |
| FreeCalcium | 3,881 | 1.11 (1.07, 1.15) | 1.10 (1.06, 1.14) | | 0.001 | 0.002 |
| Unknown |  | 264 | 23 | |  |  |
| PT | 4,167 | 15.2 (13.4, 18.6) | 20.6 (16.7, 25.5) | | <0.001 | <0.001 |
| Unknown |  | 1 | 0 | |  |  |
| APTT | 4,167 | 40 (31, 55) | 46 (38, 56) | | <0.001 | <0.001 |
| Unknown |  | 1 | 0 | |  |  |
| INR | 4,167 | 1.38 (1.22, 1.71) | 1.90 (1.51, 2.38) | | <0.001 | <0.001 |
| Unknown |  | 1 | 0 | |  |  |
| BilirubinTotal | 4,115 | 0.8 (0.4, 1.6) | 5.0 (1.8, 13.3) | | <0.001 | <0.001 |
| Unknown |  | 52 | 1 | |  |  |
| ALT | 4,124 | 41 (20, 111) | 49 (25, 135) | | <0.001 | 0.001 |
| Unknown |  | 42 | 2 | |  |  |
| AST | 4,131 | 56 (30, 159) | 98 (54, 238) | | <0.001 | <0.001 |
| Unknown |  | 36 | 1 | |  |  |
| UreaNitrogen | 4,168 | 37 (26, 53) | 40 (27, 53) | | 0.066 | 0.08 |
| Creatinine | 4,168 | 1.63 (1.18, 2.43) | 1.80 (1.25, 2.63) | | 0.007 | 0.009 |
| LD | 3,803 | 361 (262, 588) | 314 (240, 459) | | <0.001 | <0.001 |
| Unknown |  | 344 | 21 | |  |  |
| HR | 4,168 | 88 (79, 97) | 88 (80, 97) | | 0.68 | 0.72 |
| NBPS | 4,120 | 115 (106, 126) | 110 (101, 119) | | <0.001 | <0.001 |
| Unknown |  | 42 | 6 | |  |  |
| NBPD | 4,120 | 62 (56, 69) | 60 (54, 66) | | <0.001 | <0.001 |
| Unknown |  | 42 | 6 | |  |  |
| NBPM | 4,120 | 75 (69, 82) | 72 (66, 79) | | <0.001 | <0.001 |
| Unknown |  | 42 | 6 | |  |  |
| RR | 4,168 | 20.7 (18.5, 23.0) | 19.7 (17.4, 22.1) | | <0.001 | <0.001 |
| SpO2 | 4,168 | 96.79 (95.68, 97.73) | 96.68 (95.52, 97.75) | | 0.11 | 0.13 |
| Temperature | 4,112 | 98.40 (97.98, 98.89) | 98.26 (97.86, 98.72) | | <0.001 | <0.001 |
| Unknown |  | 54 | 2 | |  |  |
| Hypertension | 4,168 |  |  | | 0.003 | 0.004 |
| Absent |  | 2,318 (65%) | 428 (71%) | |  |  |
| Present |  | 1,249 (35%) | 173 (29%) | |  |  |
| DiabetesMellitusType2 | 4,168 |  |  | | <0.001 | 0.001 |
| Absent |  | 2,269 (64%) | 424 (71%) | |  |  |
| Present |  | 1,298 (36%) | 177 (29%) | |  |  |
| HeartFailure | 4,168 |  |  | | <0.001 | <0.001 |
| Absent |  | 1,894 (53%) | 460 (77%) | |  |  |
| Present |  | 1,673 (47%) | 141 (23%) | |  |  |
| MyocardialInfarction | 4,168 |  |  | | <0.001 | <0.001 |
| Absent |  | 2,901 (81%) | 555 (92%) | |  |  |
| Present |  | 666 (19%) | 46 (7.7%) | |  |  |
| MetastaticTumor | 4,168 |  |  | | <0.001 | <0.001 |
| Absent |  | 2,992 (84%) | 567 (94%) | |  |  |
| Present |  | 575 (16%) | 34 (5.7%) | |  |  |
| ChronicKidneyDisease | 4,168 |  |  | | <0.001 | <0.001 |
| Absent |  | 2,407 (67%) | 479 (80%) | |  |  |
| Present |  | 1,160 (33%) | 122 (20%) | |  |  |
| AcuteRenalFailure | 4,168 |  |  | | >0.99 | >0.99 |
| Absent |  | 26 (0.7%) | 4 (0.7%) | |  |  |
| Present |  | 3,541 (99%) | 597 (99%) | |  |  |
| Hepatitis | 4,168 |  |  | | <0.001 | <0.001 |
| Absent |  | 3,452 (97%) | 393 (65%) | |  |  |
| Present |  | 115 (3.2%) | 208 (35%) | |  |  |
| Tuberculosis | 4,168 |  |  | | 0.99 | >0.99 |
| Absent |  | 3,425 (96%) | 577 (96%) | |  |  |
| Present |  | 142 (4.0%) | 24 (4.0%) | |  |  |
| Pneumonia | 4,168 |  |  | | 0.29 | 0.34 |
| Absent |  | 2,105 (59%) | 341 (57%) | |  |  |
| Present |  | 1,462 (41%) | 260 (43%) | |  |  |
| Stroke | 4,168 |  |  | | <0.001 | <0.001 |
| Absent |  | 3,257 (91%) | 580 (97%) | |  |  |
| Present |  | 310 (8.7%) | 21 (3.5%) | |  |  |
| Hyperlipidemia | 4,168 |  |  | | <0.001 | <0.001 |
| Absent |  | 2,157 (60%) | 509 (85%) | |  |  |
| Present |  | 1,410 (40%) | 92 (15%) | |  |  |
| COPD | 4,168 |  |  | | <0.001 | <0.001 |
| Absent |  | 3,179 (89%) | 563 (94%) | |  |  |
| Present |  | 388 (11%) | 38 (6.3%) | |  |  |
| SIRS | 4,168 |  |  | | 0.66 | 0.71 |
| Absent |  | 3,527 (99%) | 593 (99%) | |  |  |
| Present |  | 40 (1.1%) | 8 (1.3%) | |  |  |
| Sepsis | 4,168 |  |  | | <0.001 | <0.001 |
| Absent |  | 2,919 (82%) | 440 (73%) | |  |  |
| Present |  | 648 (18%) | 161 (27%) | |  |  |
| MODS | 4,168 |  |  | | 0.78 | 0.82 |
| Absent |  | 3,530 (99%) | 594 (99%) | |  |  |
| Present |  | 37 (1.0%) | 7 (1.2%) | |  |  |
| SOFA | 4,168 | 8 (5, 11) | 11 (8, 14) | | <0.001 | <0.001 |
| APSIII | 4,168 | 59 (46, 77) | 72 (57, 89) | | <0.001 | <0.001 |
| SAPSII | 4,168 | 47 (38, 58) | 48 (40, 57) | | 0.037 | 0.047 |
| OASIS | 4,168 | 37 (32, 44) | 37 (31, 43) | | 0.42 | 0.47 |
| GCS | 4,157 | 15 (14, 15) | 15 (13, 15) | | 0.017 | 0.022 |
| Unknown |  | 11 | 0 | |  |  |
| Charlson | 4,168 | 6 (4, 8) | 6 (4, 8) | | 0.54 | 0.59 |
| ^1^ Median (IQR); n (%) |  |  |  | |  |  |
| ^2^ Wilcoxon rank sum test; Pearson's Chi-squared test; Fisher's exact test |  |  |  | |  |  |
| ^3^ False discovery rate correction for multiple testing |  |  |  | |  |  |
